# Age‐specific clinicopathological characteristics and prognostic analysis of neuroendocrine carcinomas of the gallbladder

**DOI:** 10.1002/cam4.4463

**Published:** 2021-11-28

**Authors:** Zhiwei Zhang, Tong Guo, Xiaorui Huang, Peng Xie, Lu Wang, Yahong Yu

**Affiliations:** ^1^ Department of Biliopancreatic Surgery Tongji Hospital Tongji Medical College Huazhong University of Science and Technology Wuhan China

**Keywords:** adenocarcinoma, gallbladder cancer, neuroendocrine carcinoma, overall survival rate, propensity score matching

## Abstract

**Background:**

We have limited information about neuroendocrine carcinoma (NEC) of the gallbladder. The purpose of this paper is to compare clinical and pathological features between different age groups and prognostic factors for gallbladder NEC and how it differs from adenocarcinoma (ADC) of the gallbladder.

**Patients and methods:**

This study included 28 gallbladder NEC patients and 137 ADC patients whose clinical characteristics and pathological findings were retrospectively collected. Propensity score matching and Cox regression analysis were used for the analysis of prognostic factors.

**Results:**

We divided NEC patients into two groups based on the age more than or less than 60 years. Most of the NEC patients less than 60 years old complained of abdominal pain or discomfort (*p* = 0.038), and more younger patients accepted adjuvant therapy (*p* = 0.020) than older patients did. CD56 was positive in all patients more than 60 years old, which is significantly higher than that of younger patients (*p* = 0.039). The mean age was similar between NEC and ADC patients. After eliminating confounding factors between NEC and ADC patients, the overall survival rates were still lower in NEC patients. Univariate analysis extracted six possible risk factors. Multivariate analysis indicated that surgery type, tumor size, and existence of gallstones were independent prognostic factors.

**Conclusion:**

The overall survival of gallbladder NEC is not associated with age. In this study, surgical method and tumor size were found to be independent risk factors for NECs. In addition, NEC patients have a worse prognosis than ADC patients with similar clinical and pathological features.

## Graphical abstract

The overall survival of gallbladder neuroendocrine carcinoma (NEC) is not associated with age. In this study, surgical method and tumor size were found to be independent risk factors for NECs. In addition, NEC patients have a worse prognosis than adenocarcinoma patients with similar clinical and pathological features.

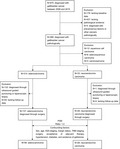

## 1 INTRODUCTION

In the clinical diagnosis and treatment process, there is an impression that tumors occurring in younger people seem to be more aggressive and the patient overall survival time tends to be shorter than for the same type of tumor in older people. A previous study about biliary tract neuroendocrine carcinoma (NEC) also indicated that age was an independent prognostic factor.^1^

The incidence of NEC of the gallbladder is very low. Although gallbladder cancer is the sixth most common cancer in the digestive system, NECs of the gallbladder account for only about 2% of all gallbladder cancers and approximately 0.5% of all NECs.^2^, ^3^

The pathogenesis of this disease is still not clear. Because neuroendocrine cells do not exist in the gallbladder, it is thought that NECs of the gallbladder are transformed from adenocarcinoma (ADC) with chronic inflammation as an important trigger.^4^ It is known that pancreaticobiliary maljunction is an inducement for gallbladder ADC. There have also been cases reported of gallbladder NECs accompanied by pancreaticobiliary maljunction.^5^, ^6^

Previous studies have suggested that gallbladder NECs, like gallbladder ADCs, are more common in females.^7^ At the time of diagnosis, most of them were at advanced stages. Considering the subtypes, it is conventionally thought that most neuroendocrine neoplasms (NENs) of the gallbladder are NECs, and that NECs are predominantly small cell neuroendocrine carcinomas (SCNECs).^8^, ^9^, ^10^, ^11^ Some case reports and case series published recently reported that the proportions of SCNEC and large cell neuroendocrine carcinoma (LCNEC) of gallbladder are similar.^7^, ^12^

Compared with gallbladder ADCs, the general demographic features of gallbladder NECs such as age, sex, and BMI are similar.^13^ No presurgical clinical feature has shown any significant differences between NECs and ADCs, while almost all the studies have suggested that NECs have a poorer prognosis than that of ADCs.^13^, ^14^

We retrospectively collected clinical and histological characteristics of gallbladder cancer patients admitted to Tongji Hospital and Wuhan Union Hospital between 2009 and 2019. Information was collected on a total of 28 patients diagnosed with NEC and 137 patients diagnosed with ADC. The NEC patients were divided into two groups, those older than and younger than age 60 years (patients aged 60 were in the older group). Clinical characteristics and pathological features between different age groups were compared. Twenty‐two factors were included in a univariate analysis. Variables with a *p* value less than 0.1 were selected for multivariate analysis. The Kaplan–Meier survival curves of different variables were drawn. Then we used propensity score matching (PSM) with a 1:2 ratio to screen out 56 ADC patients and compared their baseline information with NEC patients. No significant difference was observed between them after PSM.

The histological classification of NEC involved in this study was based on the World Health Organization (WHO) criteria updated in 2019. The main change in these criteria is that NEC is no longer considered the same as a G3 neuroendocrine tumor (NET). All the NETs are well differentiated and NECs are poorly differentiated. Mixed NENs are classified by a new term, mixed neuroendocrine non‐neuroendocrine neoplasms (MiNENs).^15^ The TNM (primary tumor, regional lymph nodes, and distant metastasis) staging of NECs and ADCs in this study was based on the eighth Edition of the American Joint Committee on Cancer (AJCC) Cancer Staging of Pancreas and Hepatobiliary Cancers.^16^

This article aims at further understanding of the characteristics of gallbladder NECs and how they differ from gallbladder ADCs.

## 2 PATIENTS AND METHODS

### 2.1 Patients

**FIGURE 1:**
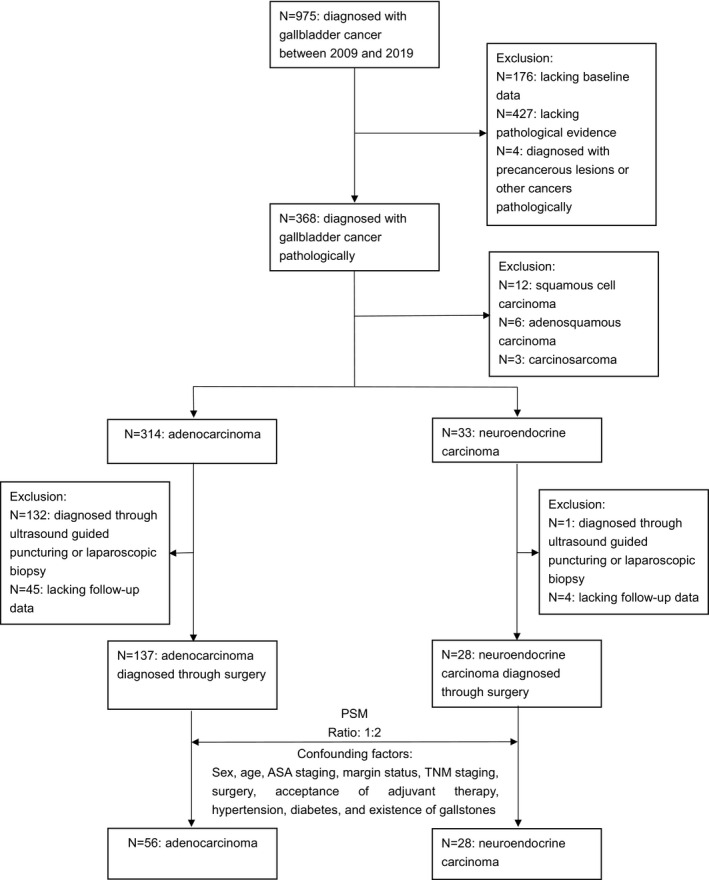
Flowchart of case selection. Situation of patients who are pathologically diagnosed with precancerous lesions or other cancers are as follows: Two patients are diagnosed with cholangiocellular carcinoma pathologically; one patient is diagnosed with low‐grade intraepithelial neoplasm pathologically; one patient is diagnosed with papillary adenoma pathologically

We retrospectively collected data on patients who had undergone surgery and were diagnosed with gallbladder cancer in Tongji Hospital and Wuhan Union Hospital between 2009 and 2019. The process of case selection is presented in Figure 1. All surgeries were conducted by experienced surgeons and pathological diagnoses were confirmed by expert pathologists at the Pathology Department of these two hospitals. Patients were excluded according to the following criteria: (1) patients with incomplete medical information or follow‐up data; (2) the primary tumor site was proved not to be in the gallbladder by pathology; (3) the pathological result was precancerous lesions or other subtypes of gallbladder cancer; and (4) pathological evidence was acquired through ultrasound‐guided puncturing or laparoscopic biopsy. NECs were diagnosed according to the following pathological criteria: (1) microscopic image displayed morphological features of NEC and (2) positive results for at least one kind of general neuroendocrine marker, including synaptophysin (Syn), chromogranin A (CgA), and CD56. Finally, 137 cases of gallbladder ADC and 28 cases of gallbladder NEC were selected for study.

The surgical method for gallbladder cancer patients varies depending on TNM stage. At the two hospitals involved in this study, patients could undergo surgery if the following conditions were met: (1) physical condition of the patient allowed radical surgery; (2) preoperative imaging showed a mass in the gallbladder, with or without hepatic invasion; (3) trunk of the portal vein or common hepatic artery was not invaded; (4) less than two metastatic tumors to the liver, and the metastatic tumor was suitable for surgical resection or microwave ablation after multiple disciplinary assessment; (5) indocyanine green test showing sufficient residual liver volume to compensate; and (6) no peritoneal metastasis found at surgery. The American Society of Anesthesiologists (ASA) classification was used for the assessment of patients’ physical condition. Patients scoring less than or equal to three were considered suitable for surgery. We divided patients into three groups in accordance with volume of hepatectomy. Patients in group 1 underwent cholecystectomy with or without common bile duct exploration, gastrojejunostomy, or cholangioenterostomy. Group 2 had only wedge resection of the liver. Group 3 had extensive liver surgeries such as resection of segments IVb and V of liver, right hepatectomy, partial hepatectomy accompanied by microwave ablation or left lateral lobectomy of the liver or right hemicolectomy, or hepatopancreaticoduodenectomy. In this study, microwave ablation was classified as having a negative margin.

The following information was retrospectively collected: sex, age, chief complaint, background disease, ASA score, tumor size, serum tumor marker, treatment, acceptance of adjuvant therapy, and pathological features, such as AJCC staging, margin status, etc. Then we divided the NEC cases into two groups according to age, over or under age 60.

The institutional review board of the Tongji Hospital and Wuhan Union Hospital, Tongji Medical College, Huazhong University of Science and Technology approved this study.

### 2.2 Pathological classification and staging

We referred to the eighth Edition of the AJCC Cancer Staging of Pancreas and Hepatobiliary Cancers and 2019 WHO classification of tumors of the digestive system in this study. NENs are divided into NETs, NECs, and MiNENs. NETs are well‐differentiated NETs that are divided into three grades based on mitotic rate and Ki‐67 index: G1 (low grade), defined as having a mitotic rate <2 per 2 mm^2^ and/or Ki‐67 <3%; G2 (intermediate grade), mitotic rate 2–20 per 2 mm^2^ and/or Ki‐67 3%–20%; and G3 (high grade), mitotic rate >20 per 2 mm^2^ and/or Ki67 >20%. In this edition, NEC, which is poorly differentiated, is recognized as different than a G3 NET. NECs include the small cell type and large cell type with a mitotic rate >20 per 2 mm^2^ and/or Ki67 >20%. MiNENs are mostly poorly differentiated in both neuroendocrine components and non‐neuroendocrine components, and each component should get graded separately. Differing from the previous edition, gastrointestinal and pancreatic NENs now use the same classification criteria.

### 2.3 Propensity score matching

The PSM method was implemented through R software for Windows (version 4.4.4). We chose the “MatchIt” package for PSM and “tableone” package for estimating standardized mean difference (SMD) values. The method was nearest neighbor matching and the distance was prop.score. There were 28 NEC patients and 137 ADC patients included for propensity scoring in a 1:2 ratio. Sex, age, ASA score, margin status, TNM staging, surgery, acceptance of adjuvant therapy, hypertension, diabetes, and the existence of gallstones were considered as confounding factors in the process. Finally, 56 ADC patients were selected.

### 2.4 Statistical analysis

The continuous parameters were analyzed with an independent samples *t*‐test or the Wilcoxon rank‐sum test and expressed as mean ± SD. Categorical variables were subjected to the Fisher's exact test or the chi‐squared test. The Kaplan–Meier method was used to estimate the overall survival rates. Univariate and multivariate analyses were implemented using Cox regression analysis and variates whose *p* value was less than 0.1 in univariate analysis were included in multivariate analysis. The statistics above were analyzed using SPSS 23.0 software (IBM Corporation). PSM was performed with R for windows 4.0.4. A *p* < 0.05 was considered as statistically significant.

## 3 RESULTS

### 3.1 Clinical characteristics of patients

Data on the clinical characteristics of NEC patients are presented in Table 1. The median age of patients diagnosed with NEC of the gallbladder was 60 years old, ranging from 34 to 85 years. Patients were predominantly female (64.3%), and more so in younger patients (76.9% vs. 53.3%). However, no significant sex difference was found between younger and older patients. The most common clinical symptom was abdominal pain or discomfort (20 patients, 71.4%). Twelve younger patients (92.3%) complained of abdominal pain and only half of older patients had this chief complaint (*p* = 0.022). Pain mainly occurred in the right upper quadrant. Two patients presented with fatigue. One of them presented jaundice at the same time, which could be the possible cause of the fatigue, and the patient's NEC was stage IIIA. Another patient complained of lack of strength for 2 years and whose tumor was classified as stage IIB. In those patients with no symptoms, tumors were found upon physical examination: two were hepatic masses and others were neoplasms in the gallbladder. The mean tumor size was larger in younger patients, but no significant difference was found (6.5 ± 3.9 vs. 4.4 ± 2.2, *p* = 0.089). Gallstones existed in 35.7% of all cases, and the rate was slightly higher in older patients (40% vs. 30.8%). There was no significant difference in underlying diseases, ASA score, neutrophil‐to‐lymphocyte ratio (NLR), carcinoembryonic antigen, carbohydrate antigen 19‐9 (CA19‐9), or carbohydrate antigen 125 (CA‐125) between younger and older patients.

**TABLE 1:** Clinical features of NEC patients

Variables^d^	Total (*N* = 28)	<60 years (*N* = 13)	≥60 years (*N* = 15)	*p* value
Age, median (range) [year]	60 (34–85)	49 (34–56)	67 (60–85)	
Sex, *N* (%)				0.254
Male	10 (35.7)	3 (23.1)	7 (46.7)	
Female	18 (64.3)	10 (76.9)	8 (53.3)	
Clinical symptom, *N* (%)^a^				
Abdominal pain or discomfort	20 (71.4)	12 (92.3)	8 (53.3)	0.038*
Jaundice	4 (14.3)	1 (7.7)	3 (20.0)	0.600
Fatigue	2 (7.1)	0	2 (13.3)	0.484
Asymptomatic	4 (14.3)	1 (7.7)	3 (20.0)	
Existence of gallstone, *N* (%)				0.705
Yes	10 (35.7)	4 (30.8)	6 (40.0)	
No	18 (64.3)	9 (69.2)	9 (60.0)	
Underlying diseases, *N* (%)^b^				
Hypertension	6 (21.4)	1 (7.7)	5 (33.3)	0.173
Diabetes mellitus	3 (10.7)	0	3 (20.0)	0.226
ASA score, *N* (%)				0.204
1	2 (7.1)	1 (7.7)	1 (6.7)	
2	20 (71.4)	11 (84.6)	9 (60.0)	
3	6 (21.4)	1 (7.7)	5 (33.3)	
Surgery, *N* (%)^c^				0.351
Group 1	7 (25.0)	2 (15.4)	5 (33.3)	
Group 2	12 (42.9)	5 (38.5)	7 (46.7)	
Group 3	9 (32.1)	6 (46.2)	3 (20.0)	
Acceptance of adjuvant therapy				0.020*
Yes	12 (42.9)	9 (69.2)	3 (20.0)	
No	16 (57.1)	4 (30.8)	12 (80.0)	
Tumor size [cm]	5.4 ± 3.3	6.5 ± 3.9	4.4 ± 2.2	0.089
NLR	7.4 ± 10.1 (*N* = 27)	5.9 ± 6.2 (*N* = 12)	8.7 ± 12.6 (*N* = 15)	0.648
CEA [ng/ml]	4.9 ± 10.2 (*N* = 21)	7.4 ± 14.7 (*N* = 10)	2.7 ± 1.3 (*N* = 11)	0.918
CA19‐9 [U/ml]	157.5 ± 341.1 (*N* = 23)	84.5 ± 130.9 (*N* = 10)	213.8 ± 438.8 (*N* = 13)	0.738
CA‐125 [U/ml]	31.5 ± 45.7 (*N* = 14)	27.5 ± 14.6 (*N* = 5)	33.7 ± 57.2 (*N* = 9)	0.817

Abbreviations: ASA, American Society of Anesthesiologists; CA‐125, carbohydrate antigen 125; CA19‐9, carbohydrate antigen 19‐9; CEA, carcinoembryonic antigen; NEC, neuroendocrine carcinoma; NLR, neutrophil‐to‐lymphocyte ratio.

^a^ Three patients had two symptoms simultaneously. One of them (60 years old) had the symptom of fatigue and jaundice and the other two (54 and 43 years old) had the symptom of abdominal pain and jaundice.

^b^ Two patients have hypertension and diabetes mellitus at the same time.

^c^ Patients in group 1 underwent cholecystectomy with or without common bile duct exploration, gastrojejunostomy, or cholangioenterostomy. Group 2 had only wedge resection of the liver. Group 3 had extensive liver surgeries such as resection of segments IVb and V of liver, right hepatectomy, partial hepatectomy accompanied by microwave ablation or left lateral lobectomy of the liver or right hemicolectomy, or hepatopancreaticoduodenectomy.

^d^ The statistics of NLR, CEA, CA19‐9, and CA‐125 were available in 27, 21, 23, and 14 patients, respectively.

* Statistically significant.

All the gallbladder NEC patients enrolled in this study had undergone surgery and were divided into three surgical groups. All patients in group 1 achieved negative margins while the proportions in group 2 and group 3 were 58.3% and 77.8%, respectively. In total, seven patients did not have a negative margin. According to pathology, five of them were stage IV and the rest turned out to be stage III tumors. The surgeries they underwent included partial hepatectomy with or without choledochectomy or hepatopancreaticoduodenectomy. The proportion of patients who accepted adjuvant therapies accounted for 42.9% of all the cases. Most stage II and stage III patients chose not to undergo adjuvant therapy. The median ages of patients who received adjuvant therapy or not were 51.5 and 66.0 years old, respectively. Platinum‐based chemotherapy was implemented in most patients who received adjuvant therapy.

### 3.2 Pathological and immunohistochemical features

The histological features of NEC patients are presented in Table 2. All cases were poorly differentiated and none of them showed acute or chronic cholecystitis. Most of the tumors in this study were stage T3 and the percentage of stage IVB was obviously higher in younger patients (61.5% vs. 33.3%). CD56 was positive in 21 of 25 patients, and it was positive in all elderly patients (13 of 13), which was significantly higher than that of younger patients (*p* = 0.039). Synaptophysin and chromogranin A were positive in 26 and 21 of all patients, respectively. The Ki‐67 index was available for all patients and was ≥70% in more than half of them. There was no statistically significant difference between different age groups with respect to AJCC staging and margin status.

**TABLE 2:** Histological features of NEC patients

Variables	Total (*N* = 28)	<60 years (*N* = 13)	≥60 years (*N* = 15)	*p* value
AJCC staging, *N* (%)				0.522
Stage IIA	2 (7.1)	0	2 (13.3)	
Stage IIB	4 (14.3)	1 (7.7)	3 (20.0)	
Stage IIIA	3 (10.7)	1 (7.7)	2 (13.3)	
Stage IIIB	5 (17.9)	3 (23.1)	2 (13.3)	
Stage IVA	1 (3.6)	0	1 (6.7)	
Stage IVB	13 (46.4)	8 (61.5)	5 (33.3)	
Margin status, *N* (%)^a^				0.670
Positive	7 (25.0)	4 (30.8)	3 (20.0)	
Negative	21 (75.0)	9 (69.2)	12 (80.0)	
CD56, *N* (%)^b^				0.039*
Positive	21 (84.0)	8 (66.7)	13 (100.0)	
Negative	4 (16.0)	4 (33.3)	0	
Syn, *N* (%)				0.484
Positive	26 (92.9)	13 (100.0)	13 (86.7)	
Negative	2 (7.1)	0 (0.0)	2 (13.3)	
CgA, *N* (%)				>0.999
Positive	21 (75.0)	10 (76.9)	11 (73.3)	
Negative	7 (25.0)	3 (23.1)	4 (26.7)	
Ki‐67, median (range)	75 (20–97)	70 (20–97)	80 (20–97)	0.966

Abbreviations: AJCC, American Joint Committee on Cancer; CD56, cluster of differentiation 56; CgA, chromogranin A; NEC, neuroendocrine carcinoma; Syn, synaptophysin.

^a^ Microwave ablation was considered as the same effect of R0 resection.

^b^ The statistics of CD56 were available in 25 patients (12 patients <60 years and 13 patients ≥60 years).

* Statistically significant.

### 3.3 Clinical outcomes

**FIGURE 2:**
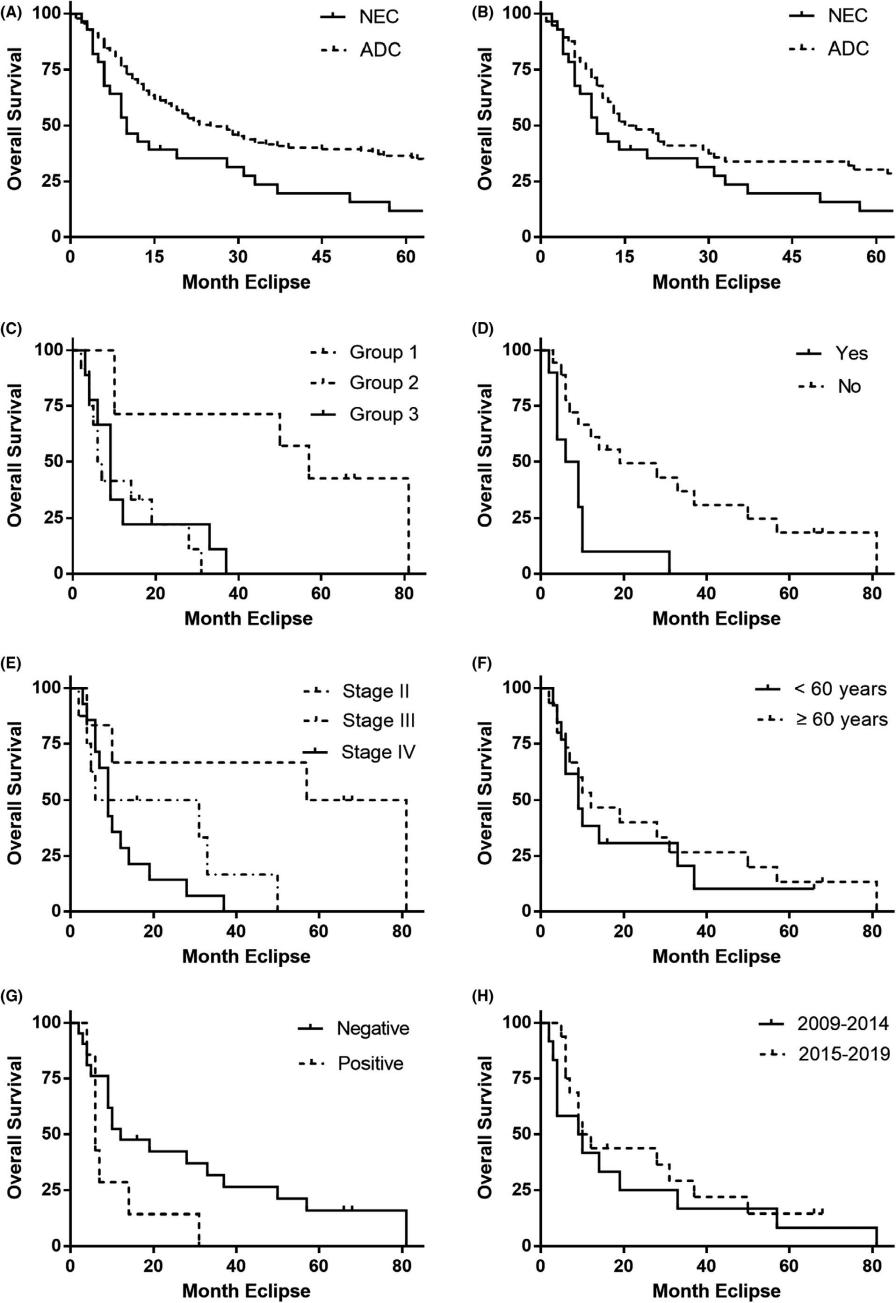
The Kaplan–Meier survival curves. There is significant difference in overall survival (OS) between neuroendocrine carcinoma (NEC) and adenocarcinoma (ADC) patients either before (A) or after (B) propensity score matching. In NEC patients, surgery (C) and existence of gallstone (D) are recognized as independent risk factors. Considering TNM staging (E), patients with stage II tumors live significantly longer than those with stage III and IV tumors. Age (F), margin status (G), and year of operation (H) are not associated with the OS of NEC

The outcomes of the Kaplan–Meier survival analysis are presented in Figure 2. Possible risk factors identified by univariate analysis (*p* < 0.1) were extracted and included in multivariate Cox regression analysis for independent risk factors (Table 3). Surgery type, tumor size, and existence of gallstone were recognized as independent risk factors after multivariate analysis. The relationship between surgery and TNM staging or margin status is presented in Table 4. It is obvious that patients who had more extensive surgeries were in advanced stages (*p* = 0.001).

**TABLE 3:** Univariate analysis and multivariate analysis of NEC patients

Variables		Univariate analysis		Multivariate analysis	
		*p* value	HR (95% CI)	*p* value	HR (95% CI)
Age	≥60 years vs. <60 years	0.600	0.804 (0.357–1.813)		
Sex	Male vs. female	0.696	0.844 (0.359–1.981)		
Year of operation^a^	Group 2 vs. Group 1	0.438	0.727 (0.325–1.628)		
Abdominal pain or discomfort	Yes vs. no	0.994	1.003 (0.413–2.438)		
Jaundice	Yes vs. no	0.990	1.007 (0.343–2.957)		
Existence of gallstone	Yes vs. no	0.008*	3.428 (1.374–8.552)	0.043*	3.554 (1.004–12.099)
Hypertension	Yes vs. no	0.667	1.242 (0.462–3.340)		
ASA score	2 vs. 1	0.567	0.647 (0.146–2.869)		
	3 vs. 1	0.908	0.909 (0.179–4.612)		
Surgery^b^	Group 2 vs. Group 1	0.006*	9.505 (1.916–47.145)	0.046*	6.929 (1.036–46.364)
	Group 3 vs. Group 1	0.010*	7.905 (1.653–37.804)	0.037*	6.741 (1.117–40.677)
Receipt of adjuvant therapy	Yes vs. no	0.777	0.888 (0.390–2.021)		
Tumor size		0.006*	1.221 (1.058–1.409)	0.006*	1.267 (1.070–1.500)
NLR (*N* = 27)		0.490	0.982 (0.932–1.034)		
CEA (*N* = 21)		0.134	1.036 (0.989–1.805)		
CA19‐9 (*N* = 23)		0.652	1.000 (0.999–1.002)		
CA‐125 (*N* = *N* = 14)		0.638	0.997 (0.986–1.009)		
AJCC staging	Stage III vs. II	0.041*	5.339 (1.070–26.647)		
	Stage IV vs. II	0.010*	7.983 (1.627–39.165)		
Margin status	R0 vs. R1	0.073	0.422 (0.165–1.083)		
Liver metastasis	Yes vs. no	0.153	1.875 (0.792–4.441)		
CD56 (*N* = 25)	Positive vs. negative	0.887	0.914 (0.266–3.143)		
Syn	Positive vs. negative	0.513	1.627 (0.378–6.999)		
CgA	Positive vs. negative	0.558	1.321 (0.521–3.351)		
Ki‐67		0.035*	0.981 (0.963–0.999)		

Abbreviations: AJCC, American Joint Committee on Cancer; ASA, American Society of Anesthesiologists; CA‐125, carbohydrate antigen 125; CA19‐9, carbohydrate antigen 19‐9; CD56, cluster of differentiation 56; CEA, carcinoembryonic antigen; CgA, chromogranin A; CI, confidence interval; HR, hazard ratio; NEC, neuroendocrine carcinoma; NLR, neutrophil‐to‐lymphocyte ratio; Syn, synaptophysin.

^a^ Group 1: 2009–2014; Group 2: 2015–2019.

^b^ Patients in group 1 underwent cholecystectomy with or without common bile duct exploration, gastrojejunostomy, or cholangioenterostomy. Group 2 had only wedge resection of the liver. Group 3 had extensive liver surgeries such as resection of segments IVb and V of liver, right hepatectomy, partial hepatectomy accompanied by microwave ablation or left lateral lobectomy of the liver or right hemicolectomy, or hepatopancreaticoduodenectomy.

* Statistically significant.

**TABLE 4:** Characteristics of different surgical groups of NEC patients

Variables	Surgery^a^			*p* value
	Group 1, *N* = 7	Group 2, *N* = 12	Group 3, *N* = 9	
AJCC staging, *N* (%)				0.001*
Stage II	5 (71.4)	1 (8.3)	0	
Stage III	1 (14.3)	6 (50.0)	1 (11.1)	
Stage IV	1 (14.3)	5 (41.7)	8 (88.9)	
Margin status, *N* (%)				0.156
Positive	0	5 (41.7)	2 (22.2)	
Negative	7 (100)	7 (58.3)	9 (77.8)	

Abbreviations: AJCC, American Joint Committee on Cancer; NEC, neuroendocrine carcinoma.

^a^ Patients in group 1 underwent cholecystectomy with or without common bile duct exploration, gastrojejunostomy, or cholangioenterostomy. Group 2 had only wedge resection of the liver. Group 3 had extensive liver surgeries such as resection of segments IVb and V of liver, right hepatectomy, partial hepatectomy accompanied by microwave ablation or left lateral lobectomy of the liver or right hemicolectomy, or hepatopancreaticoduodenectomy.

* Statistically significant.

Given the fact that only a few patients live more than 3 years, the 1‐, 2‐ and 3‐year survival rates were calculated and are presented in Table 5. The median survival time in younger patients was 9 months, while in the elderly it was 12 months, but the differences between them were not significant (*p* = 0.467). Those patients who underwent adjuvant therapy had a longer median survival time (15 months vs. 9.5 months, *p* = 0.777).

**TABLE 5:** Overall survival rates before and after PSM

	1‐year survival rates			2‐year survival rates			3‐year survival rates		
	NEC (%)	ADC before PSM (%)	ADC after PSM (%)	NEC (%)	ADC before PSM (%)	ADC after PSM (%)	NEC (%)	ADC before PSM (%)	ADC after PSM (%)
AJCC staging									
Stage 0	–	100.0	–	–	100.0	–	–	100.0	–
Stage I	–	100.0	–	–	100.0	–	–	91.7	–
Stage II	66.7	89.3	100.0	66.7	82.1	87.5	66.7	71.4	75.0
Stage III	50	65.7	63.3	37.5	40.3	46.6	12.5	32.8	39.9
Stage IV	35.7	50.0	44.4	14.3	21.4	11.1	7.1	7.1	5.6
OS	46.4	70.8	62.5	32.1	50.4	41.1	21.4	41.6	34.0

Abbreviations: ADC, adenocarcinoma; AJCC, American Joint Committee on Cancer; NEC, neuroendocrine carcinoma; OS, overall survival; PSM, propensity score matching.

### 3.4 Propensity score matching

The *p* values and SMD values of confounding factors before and after PSM are presented in Table 6. Before PSM, the proportion of males was 35.7% in the NEC group and 30.7% in the ADC group; the mean age was 58.93 years in NEC (range 34–85) and 58.56 years in ADC (range 32–85). Except for TNM staging, differences in other covariates such as ASA score, margin status, surgery type, acceptance of adjuvant therapy, hypertension, diabetes mellitus, and the existence of gallstone were not significant. Distribution of TNM staging was significantly different between NEC and ADC patients (*p* = 0.011). After the propensity score procedure, no significant differences existed in baseline data, and the SMD value had been reduced to a relatively low level. However, even if other baseline characteristics were similar, the 1‐year and 3‐year overall survival rates in the ADC group were significantly higher (*p* < 0.05).

**TABLE 6:** Characteristics of gallbladder ADC and NEC patients before and after PSM

Variables	Before PSM				After PSM			
	Total (*n* = 165)	NECs (*n* = 28)	ADCs (*n* = 137)	*p* value	Total (*n* = 84)	NECs (*n* = 28)	ADCs (*n* = 56)	*p* value
Age, mean (range)	58.62 (32–85)	58.93 (34–85)	58.56 (32–85)	0.863	58.99 (32–85)	58.93 (34–85)	59.02 (32–78)	0.973
Sex, *N* (%)				0.763				>0.999
Male	52 (31.5)	10 (35.7)	42 (30.7)		29 (34.5)	10 (35.7)	19 (33.9)	
Female	113 (68.5)	18 (64.3)	95 (69.3)		55 (65.5)	18 (64.3)	37 (66.1)	
ASA score, *N* (%)				0.838				0.777
1	14 (8.5)	2 (7.1)	12 (8.8)		6 (7.1)	2 (7.1)	4 (7.1)	
2	123 (74.5)	20 (71.4)	103 (75.2)		64 (76.2)	20 (71.4)	44 (78.6)	
3	27 (16.4)	6 (21.4)	21 (15.3)		14 (16.7)	6 (21.4)	8 (14.3)	
4	1 (0.6)	0 (0.0)	1 (0.7)		–	–	–	
Surgery, *N* (%)				0.676				0.886
Group 1	49 (29.7)	7 (25.0)	42 (30.7)		23 (27.4)	7 (25.0)	16 (28.6)	
Group 2	59 (35.8)	12 (42.9)	47 (34.3)		33 (39.3)	12 (42.9)	21 (37.5)	
Group 3	57 (34.5)	9 (32.1)	48 (35.0)		28 (33.3)	9 (32.1)	19 (33.9)	
Margin status, *N* (%)				0.091				>0.999
Negative	143 (86.7)	21 (75.0)	122 (89.1)		64 (76.2)	21 (75.0)	43 (76.8)	
Positive	22 (13.3)	7 (25.0)	15 (10.9)		20 (23.8)	7 (25.0)	13 (23.2)	
AJCC staging, *N* (%)				0.011*				0.095
Stage 0	2 (1.2)	0 (0.0)	2 (1.5)		–	–	–	
Stage I	12 (7.3)	0 (0.0)	12 (8.8)		–	–	–	
Stage II	34 (20.6)	6 (21.4)	28 (20.4)		14 (16.7)	6 (21.4)	8 (14.3)	
Stage III	75 (45.5)	8 (28.6)	67 (48.9)		38 (45.2)	8 (28.6)	30 (53.6)	
Stage IV	42 (25.5)	14 (50.0)	28 (20.4)		32 (38.1)	14 (50.0)	18 (32.1)	
Acceptance of adjuvant therapy, *N* (%)				0.622				0.589
Yes	61 (37.0)	12 (42.9)	49 (35.8)		41 (48.8)	12 (42.9)	29 (51.8)	
No	104 (63.0)	16 (57.1)	88 (64.2)		43 (51.2)	16 (57.1)	27 (48.2)	
Hypertension, *N* (%)				0.899				>0.999
Yes	31 (18.8)	6 (21.4)	25 (18.2)		17 (20.2)	6 (21.4)	11 (19.6)	
No	134 (81.2)	22 (78.6)	112 (81.8)		67 (79.8)	22 (78.6)	45 (80.4)	
Diabetes mellitus, *N* (%)				>0.999				>0.999
Yes	15 (9.1)	3 (10.7)	12 (8.8)		9 (10.7)	3 (10.7)	6 (10.7)	
No	150 (90.0)	25 (89.3)	125 (91.2)		75 (89.3)	25 (89.3)	50 (89.3)	
Existence of gallstone, *N* (%)				0.391				0.937
Yes	74 (44.8)	10 (35.7)	64 (46.7)		32 (38.1)	10 (35.7)	22 (39.3)	
No	91 (55.2)	18 (64.3)	73 (53.3)		52 (61.9)	18 (64.3)	34 (60.7)	

Abbreviations: ADC, adenocarcinoma; AJCC, American Joint Committee on Cancer; ASA, American Society of Anesthesiologists; NEC, neuroendocrine carcinoma; PSM, propensity score matching; SMD, standardized mean difference.

* Statistically significant.

## 4 DISCUSSION

This study is one of the largest studies about NEC of the gallbladder in the People's Republic of China to date.^13^ We collected the clinical and pathological characteristics of patients in two tertiary hospitals retrospectively. As was the case in previous studies, patients enrolled in this study were predominantly female.^17^ Although the clinical and pathological features were similar between NEC and ADC patients after PSM, the overall survival rates were still obviously lower in NEC patients^7^, ^13^, ^14^, ^18^

NEC patients in this study were divided into two groups according to age. In younger patients, the TNM stage was relatively late, but there was no significant difference (*p* = 0.522). At the same time, more younger patients complained of abdominal pain or discomfort. We speculate that this is because the elderly care more about their physical condition and tend to have regular health examinations so that tumors may be found at a relatively early stage. However, more younger patients accepted adjuvant therapy after surgery. CD56, also called neural cell adhesion molecules (NCAM), is a group of glycoproteins used for the diagnosis of NETs. Some studies have found that CD56‐positive tumors are more invasive.^19^ NCAM was positive in all older patients in this study and was significantly higher than that in younger patients (*p* = 0.039). Two NEC patients had the chief complaint of fatigue, but no ADC patient complained of this. However, because of the first pass effect, less than 5% of gastroenteropancreatic NECs present with hormonal syndromes.^20^ Patients with advanced tumors usually have symptoms such as fatigue or weight loss. So, when a patient with a gallbladder neoplasm complains of fatigue, NEC should be considered, but this is not a strong indicator. Some case reports also reported flushing, Cushing's syndrome, or hypoglycemia as the primary clinical symptoms.^21^, ^22^, ^23^

There is no consensus on the treatment of gallbladder NECs. Surgery is the only approach that may cure this disease. Achieving R0 margin status is important for a longer overall survival time. In addition, chemotherapy and radiotherapy are worth trying.^24^ Cisplatin and etoposide are the first‐line choices for chemotherapy.^25^ Molecular targeted therapy still needs further research. Liu et al.^26^ found that the pulmonary LCNEC is the tumor most similar to gallbladder NEC, and NAB2 and RB1 were specific mutations in cases of 15 gallbladder NEC.

A study indicated that the NLR is an indicator that suggests a worse prognosis when it is high,^27^ and the high level of NLR in this study was associated with a bad prognosis. The overall survival rates of NEC patients in this study were relatively lower than in previous studies.^7^, ^13^, ^28^ Possible reasons include the high proportion of advanced AJCC stages (stage III: 28.6%; Stage IV: 50%), poor histological grade (100% poorly differentiated), low rate of adjuvant therapy (42.9%), and poor margin status (75% R0 resection).^29^, ^30^ Yan et al. reported that the median survival time of NEC patients was 20.4 months, which is longer than that of this study (10.0 months). Their AJCC staging (stage III: 46.7%; stage IV: 26.7%) and histological grade (66.7% well differentiated) were relatively better than in patients in our study, which could lead to a longer survival time.^7^, ^13^ Chen et al.^18^ reported that the median survival time in their study was 3 months, and only two of 10 patients in their study underwent radical resection and the rest got palliative therapy. From what has been discussed above, we find that radical resection with negative margins and postoperative adjuvant therapy are important for a longer survival time.

After univariate Cox regression analysis, six factors were extracted for multivariate analysis. Surgery, tumor size, and existence of gallstones were recognized as independent risk factors. Patients who received larger surgeries tended to have advanced stage tumors with a positive surgical margin. So, it is important that we find gallbladder NEC at an early stage and ensure R0 resection during surgery. More patients with larger sized tumors were at an advanced stage in this study (Table 7). Because gallstones are a trigger for gallbladder cancer, new guidelines suggest cholecystectomy to be carried out in more patients.^31^

**TABLE 7:** Relationship between tumor size and AJCC staging of NEC

AJCC staging	Total	0 < Size < 5^a^	5 < Size < 10	Size ≥ 10
Stage II	6 (21.4)	4 (30.8)	2 (14.3)	0
Stage III	8 (28.6)	5 (28.5)	3 (21.4)	0
Stage IV	14 (50.0)	4 (30.8)	9 (64.4)	1 (100.0)
*p* = 0.370				

Abbreviations: AJCC, American Joint Committee on Cancer; NEC, neuroendocrine carcinoma.

^a^ Size represents the maximum diameter of the primary tumor on cross‐section of CT or MRI.

It is difficult to differentiate NEC from ADC before surgery, and there is no standard procedure to identify gallbladder NEC. Since some studies indicated that NEC is transformed from ADC in the gallbladder, prevention seems to be important. In patients with risk factors for gallbladder cancer such as gallstones, gallbladder polyps, chronic cholecystitis, etc., gallbladder status should be monitored and surgery carried out as necessary. Ultrasound is excellent for screening. When a mass is found, computerized tomography (CT), magnetic resonance imaging (MRI), or endoscopic ultrasonography (EUS) are helpful for staging and evaluating the surgical opportunity. Some reports have presented NEC cases that may have been diagnosed preoperatively. They thought that somatostatin receptor scintigraphy or 18FDG‐PET/CT could be potential examinations that can distinguish gallbladder NEC before surgery.^32^, ^33^ A fine‐needle aspiration under endoscopic ultrasound or biliary cytology is also helpful for preoperative diagnosis.^34^ Plasma tumor markers such as chromogranin A and neuro‐specific enolase are elevated in some advanced pulmonary NECs, but their role in gastroenteropancreatic NEC and gallbladder NEC is not clear.^20^

There are limitations of our study: (1) This is a retrospective study. It is difficult to collect complete data. The prognosis of gallbladder NEC is poor and the rate of loss to follow‐up is high. (2) The sample size was relatively limited. Results may not be generalizable. (3) Up till now, no standard diagnostic criteria for gallbladder NEC have been established. Advanced clinical studies and molecular researches are needed for disease‐specific treatment.

## 5 CONCLUSION

The overall survival of gallbladder NEC is not associated with age. NEC has a poor prognosis no matter when it develops. In this study, surgical method and tumor size were found to be independent risk factors for NECs, suggesting that detection and operation in early stage are the only chance for the long‐term survival. In addition, NEC patients have a worse prognosis than ADC patients with similar clinical and pathologic features. Since early detection and diagnosis of NEC are of great significance for its prognosis, further studies should focus on differential diagnosis of NEC and ADC. And researches on the difference in molecular mechanism between NEC and ADC is urgent for better treatment effect.

## CONFLICT OF INTEREST

No benefits in any form have been received or will be received from a commercial party related directly or indirectly to the subject of this article.

## AUTHOR CONTRIBUTION

Zhiwei Zhang: Data curation, Formal analysis, Methodology, Project administration, and Writing‐original draft; Tong Guo: Investigation, Resources and Writing‐original draft; Xiaorui Huang: Validation and Software; Peng Xie: Resources and Software; Lu Wang: Visualization; Yahong Yu: Conceptualization, Supervision, Funding acquisition, and Writing‐review & editing.

## ETHICAL APPROVAL

The institutional review board of the Tongji Hospital and Wuhan Union Hospital, Tongji Medical College, Huazhong University of Science and Technology approved this study.

## Data Availability

The data that support the findings of this study are available from the corresponding author upon reasonable request.
